# General Intelligence

**Published:** 1844-07

**Authors:** 


### GENERAL INTELLIGENCE.

THE following extracts from an "Introductory Lecture to the course of Clinical Surgery in the Pennsylvania Hospital," by G. W. Norris, M. D., published in the Medical News, are commended to the attention of the profession. Seldom have more just and noble precepts been addressed by a teacher to his pupils, never perhaps, at any period of the history of our art, were they more demanded. Coming from a surgeon of so venerable an institution, whose example so perfectly illustrates his teachings, they are calculated to have a salutary influence upon the minds of students and surgeons. D. B.

"I believe, gentlemen, that a crying and increasing defect in the teaching of these times, is a neglect of the principles of surgery. Beginners in the study of our science naturally enough become weary of the detail of principles, and when opportunities offer, love to wander off and witness operations. These when determined on with judgment, while they distract the mind from tiresome rules, instruct in what is certainly a highly important part of the profession, and to a wholesome degree should be encouraged; but important and dazzling as the calmness of mind and ability to perform them well may be, it is to be remembered that their acquisition is the least part of a surgeon's education, and a necessity for their performance the opprobrium of our science. To understand the nature of the affections in which operations are demanded, and to distinguish accurately these from such as are remediable by medical means, to determine the moment when the knife should be resorted to, and under what circumstances it is to be withheld, to know precisely what should be done before an operation, and the best course of treatment after it, are the great and difficult points to which attention should at all times be principally directed. Operative surgery, much as it should be valued, is, when compared to these, of only secondary importance; and incessantly to dwell upon it and magnify its results, and consequence, is again to degrade our science to the rank which in former times it held when connected with the barbers."

"In operative medicine as in all other matters connccted with our profession, the Father of American surgery shines forth a bright example to us, and would that they whose stations give authority to their teachings, would frequently recall it to the recollections of their pupils. Justly looking upon improvements in medical surgery as infinitely more glorious than displays with the knife, he on all occasions zealously endeavored to avoid the performance of bloody operations. Fixed in his principles, no desire of notoriety, or wish to appear as an originator, ever induced him to operate in cases of doubtful utility. Candid in his opinions to the suffering on the probable benefits of operative measures, and to the profession on his results, he rose to occupy that niche in the Temple of Fame to which his countrymen have unanimously elevated him; and may we not hope that a recollection of PHYSICK's course will yet one day win back those who have strayed from the paths of legitimate surgery, impel students to eschew that desire of operative show and notoriety which is now so baneful and so common, and induce them to bestow more attention to the acquirement of the solid principles which will guide them in the treatment of disease, than to witnessing operations the actual and final results of which are not presented, and which from their nature can but in very few instances be determined by them."

---

WE cannot resist the temptation to extract from that lively and spirited periodical, the Boston Medical and Surgical Journal, the following sketch of a distinguished surgeon of New York. From its fidelity it would be easily recognized by every one acquainted with the original if the name were omitted: D. B.

"Dr. Parker is a native of Massachusetts; and for the gratification of our Yankee brethren, who may not have known him, I feel an irresistible desire to speak more particularly of him, and especially as I am sure mine were but the views and impressions received generally by the many medical gentlemen visiting that city during the past winter. Dr. P., I learned, was a graduate of Harvard, and also of the Massachusetts Medical College; of the latter about 1829 or 1830, since which time he has been constantly occupied, either in the anatomical or surgical chair of some of our medical colleges. He is now about 40, with a cheerfulness, vivacity and youthful expression that would seem to assure us that he had escaped the sorrows and internal conflicts that make visible the years of most men. Dr. P. is about, or little short of, six feet in height, of as perfect development, as it regards size, form and poportion, as is easy to conceive, and although not less so in attitude and movements, there is a careless unconsciousness of this trifling matter, that makes it of peculiar interest to the stranger. He has a dark eye, black hair, and a countenance of great animation, especially when lit up by his favorite subject—*surgery*. When we have added to these the intimate and thorough knowledge of his subject, obtained by fifteen years' zealous devotion as a lecturer and practitioner, a fine voice, easy command of language, and great facility of utterance, a discriminating mind, and a good fund of "*common sense*" withal, it will not be difficult to form some idea of him as a lecturer, or a practical surgeon. Another particular, that will be readily observed by the stranger, in the character of Professor P., is a freedom from that egotism and spirit of detraction, that has poisoned the minds and shadowed the reputations of so many able surgeons; and in its stead, he will perceive a modesty, a spirit of benevolence and sincerity, that enables the possessor as readily to discern the merits and achievements of others, and as willing to acknowledge them, as though they were his own. Hence I was enabled to account for the universal high respect and friendship entertained for him by the profession, and the readiness of his rival cotemporaries to acknowledge his merits."

---

### RUSH MEDICAL COLLEGE.

The annual course of Lectures in this Institution for the session of 1844–'45, will commence on the 1st Monday of November next.

The lectures will be delivered as follows:

On Anatomy and Surgery, by Daniel Brainard, M. D.
" Institutes and Practice of Medicine, by Austin Flint, M. D.
" Chemistry and Pharmacy, by J. V. Z. Blaney, M. D.
" Materia Medica and Therapeutics, by John McLean, M. D.
" Obstetrics and Diseases of Women and Children, by G. N. Fitch, M. D.

At a meeting of the Board of Trustees on the 24th of June ult. the degree of doctor of medicine was conferred on William M. Butterfield. The honorary degree of doctor of medicine was conferred on T. P. Whipple, of St. Charles, Kane co., Ill.

Arrangements were also made for erecting a handsome edifice for the Institution.

---

Notice to Readers and Correspondents.—We acknowledge the regular receipt (in exchange) of the Boston Medical and Surgical Journal, the Medical News, the Medical Examiner, the American Journal of Pharmacy, the Western Lancet, and the St. Louis Medical and Surgical Journal.

